# Nanometric Cutting of Silicon with an Amorphous-Crystalline Layered Structure: A Molecular Dynamics Study

**DOI:** 10.1186/s11671-017-1829-y

**Published:** 2017-01-13

**Authors:** Jinshi Wang, Fengzhou Fang, Xiaodong Zhang

**Affiliations:** State Key Laboratory of Precision Measuring Technology & Instruments, Centre of MicroNano Manufacturing Technology, Tianjin University, Tianjin, 300072 China

**Keywords:** Nanometric machining, Layered structure, Material deformation, Nano-chip formation, 68.35.bj

## Abstract

Materials with specific nanometric layers are of great value in both theoretical and applied research. The nanometric layer could have a significant influence on the response to the mechanical loading. In this paper, the nanometric cutting on the layered systems of silicon has been studied by molecular dynamics. This kind of composite structure with amorphous layer and crystalline substrate is important for nanomachining. Material deformation, stress status, and chip formation, which are the key issues in nano-cutting, are analyzed. A new chip formation mechanism, i.e., the mixture of extrusion and shear, has been observed. In addition, from the perspective of engineering, some specific composite models show the desired properties due to the low subsurface damage or large material removal rate. The results enrich the cutting theory and provide guidance on nanometric machining.

## Background

Materials with a specific layered structure such as film substrates and multilayer systems have many fascinating features. Some of these composite structures that are well designed can provide high strength and high wear resistance and are used as protective coatings [1]. To study the mechanical properties, nanoindentation [2] and nano-cutting [3] techniques are always employed. In addition, with the great advances in computer science, numerical simulation has become an essential approach to the understanding of the mechanism of material deformation under these loading conditions, especially on the nanometric scale. For example, Fang et al. studied the nanoindentation and nanoscratch on the Al/Ni multilayer by molecular dynamics method [4, 5]. The contact stiffness increased with more nickel layers in the specimen, and the specific cutting energy decreased under a high scratch speed. The dislocation evolution in the Al/Ni system was analyzed by Cao et al [6]. They found that the semi-coherent boundary hindering the dislocation movement played a key role in mechanical hardening. Sekkal et al. simulated the nanoindentation of TiC/NbC and TiC and discussed the hardness enhancement in the TiC/NbC superlattice [7]. Kizler et al. studied the effects of nanostructures including grain boundary and lattice orientation on the hardness of TiC/NbC system. It indicated that defects in the material would improve the plasticity [8].

Except for the applications of protective coatings and functional surfaces, the layered composite structure is also very common in the mechanical processes on a nanometric scale. A typical example is the phase transition of covalent crystals (e.g., Si, Ge) during the nanoindentation with fast unloading [9]. Amorphization also occurs during nano-cutting or ultra-precision turning [10]. The amorphous structure is believed to be transformed from a metallic phase (β-Sn for Si and Ge) induced by intensive hydrostatic pressure during loading [11]. Therefore, the crystal being machined in fact has an amorphous-crystalline structure instead of its original lattice. Due to different mechanical properties, the amorphous layer on the top of the crystalline bulk would affect the machining processes significantly such as ultra-precision turning, grinding, and polishing. Besides, an amorphous layer can also be used to facilitate the machining of brittle materials. For example, to control the fracture, surface amorphization, which may be achieved through ion implantation, could be performed before the mechanical processes occur. In this method, the monocrystalline workpiece is exposed to a high-energy ion beam. Large numbers of ions displace the target atoms and trigger a collision cascade [12]. As a result, the initial lattice of the workpiece is destroyed, and an amorphous layer is formed with an abundance of ions. After the implantation, the surface hardness and Young’s modulus decrease, accompanied by an increase in the ductile-brittle transition depth [13]. This method has been applied to silicon, germanium, and silicon carbide, and its validity has been proved [14, 15]. As discussed above, the amorphous-crystalline composite system has great influence on nanomachining but has not been fully researched. Thus, in this work, the nanometric cutting of this layered structure is studied systematically. The effects of various configurations of the amorphous layer on the stress state, material deformation, and chip formation during nano-cutting are analyzed. The aim of our work is also to evaluate which composite structures are preferred for nanomachining.

## Methods

Numerical simulation is conducted using the molecular dynamics (MD) method. In this section, the cutting model is presented firstly. Then, details of making the layered composite structure are reported.

### Nanometric Cutting Model

**Fig. 1:**
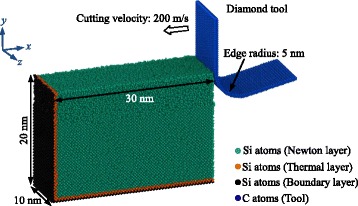
Nanometric cutting model

The cutting model consists of four parts, namely, the boundary layer, the thermal layer, the Newton layer and the tool, as illustrated in Fig. 1. The boundary layer imposes boundary constraints in the *x* and *y* directions. The thermal layer serves as a heat bath with a temperature of 293 K. Nanometric cutting is performed on the Newton layer. The models with 30, 20, and 10 nm in the *x*, *y*, and *z* dimensions consist of silicon atoms. A diamond tool with a 5-nm edge is used. Because the major task in this work is studying the material behavior in the workpiece, the tool is treated as a rigid body to reduce the computing time. The undeformed chip thickness (or the cutting depth) is 5 nm, and the tool moves opposite to the *x* axis with a velocity of 200 m/s. In addition, the nominal rake angle and clearance angle are 0° and 12°, respectively. For the monocrystalline silicon (c-Si), the tool moves along the {010} [100] orientation.

MD calculations are performed by LAMMPS [16], assisted by OVITO [17] for visualization. The nanometric cuttings are simulated in the NVE ensemble with the periodic boundary condition (PBC) in the *z* dimension. The Verlet algorithm is used to integrate the motion of Newton atoms, and the time step is 2 fs. The interactions between Si-Si and Si-C are described through a Tersoff-type potential [18]. Dynamic relaxation is performed to allow the model to be in an equilibrium state at 293 K before the cutting.

### Amorphous and Layered Model Construction

**Fig. 2:**
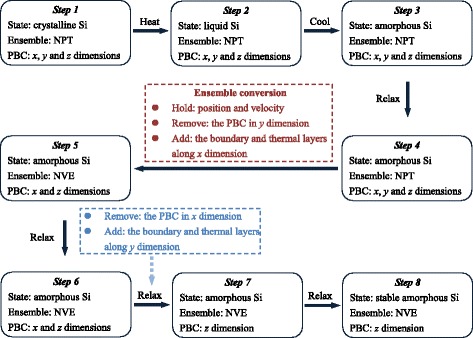
Process of amorphous model construction

**Fig. 3:**
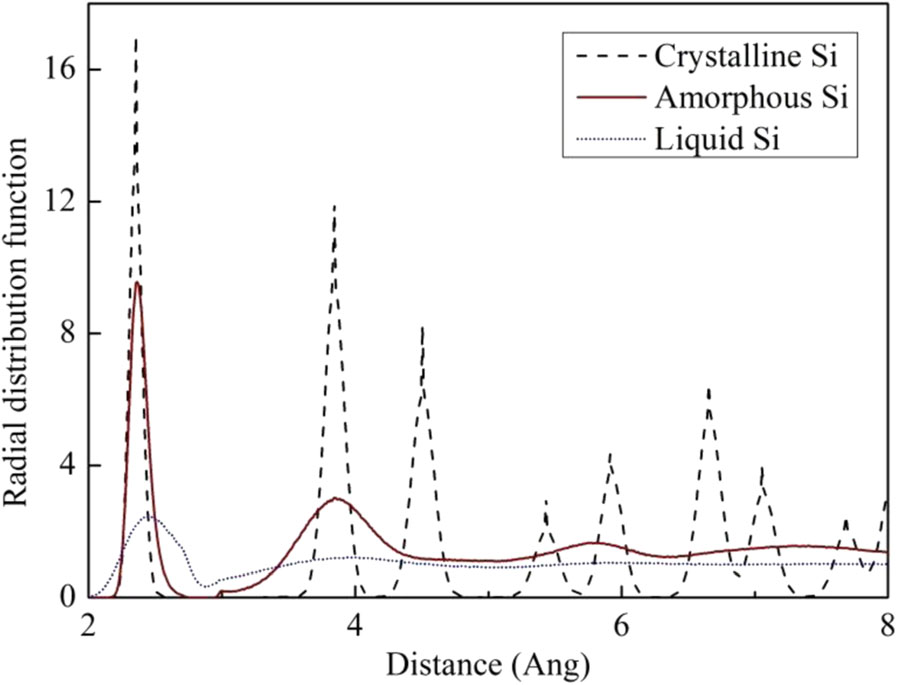
RDFs of different Si phases

The amorphous phase is prepared by quenching [19]. As the temperature rises gradually, the model eventually melts and reaches a disordered atom distribution. With a rapid cooling, the amorphous structure would be attained. Figure 2 shows the procedure for the construction of the amorphous model. During this procedure, a c-Si bulk is heated and melts at 3200 K during 10.2 ns, followed by a fast quenching in the NPT ensemble (1 ns quenching + 0.2 ns relax at 500 K). After the dynamic relaxation (6 ns) in the NVE ensemble, the temperature of the Newton layer converges to 304 K, which is only 11 K higher than the thermal layer (293 K). Figure 3 shows the radial distribution functions (RDF) of crystalline, liquid, and amorphous silicon (a-Si). Clearly, the distributed peaks indicate the order in both short and long ranges for c-Si. For a-Si, only the short range order is kept as the peaks of the first and second nearest neighbors are identical to those of c-Si. Then, for liquid Si, the RDF almost holds at 1, which represents an isotropic structure. The RDF of a-Si coincides with the RDF reported in the literature [20].

**Fig. 4:**
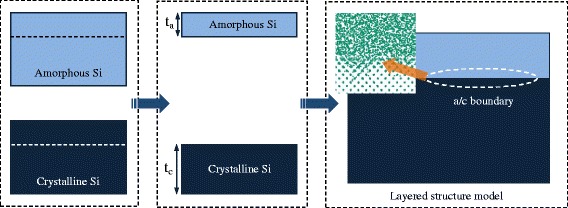
Construction of layered model

**Fig. 5:**
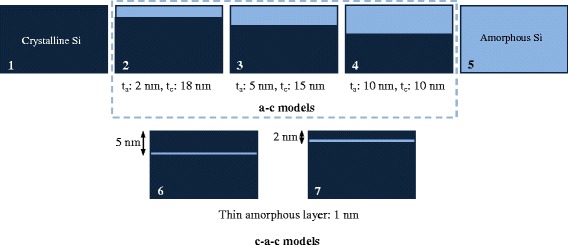
Layered structure models

Figure 4 shows the construction of the model with a layered structure. The amorphous and crystalline parts are sliced from a-Si and c-Si bulks according to specific thicknesses of t_a_ and t_c_. After the joining followed by a dynamic relaxation, the model reaches equilibrium. As a result, the relaxation coarsens the a/c boundary. Two types of layered structures are considered, as illustrated in Fig. 5. Models 1 ~ 5 with an amorphous layer on the crystalline bulk (a-c models) are used to study the effect of the amorphous layer thickness, and thin a-Si layers with a thickness of 1 nm are embedded into the c-Si bulk (c-a-c models) as models 6 ~ 7, shown in Fig. 5.

## Results and Discussion

The material behaviors of the a-c and c-a-c models are quite different and will be discussed in two separate sections.

### a-c Models

**Fig. 6:**
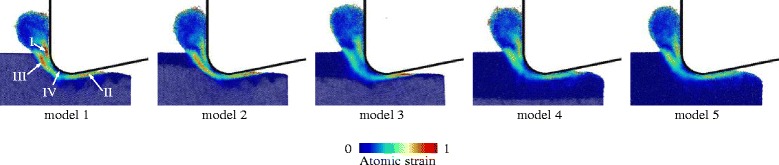
Atomic strain from model-1 to model-5

**Fig. 7:**
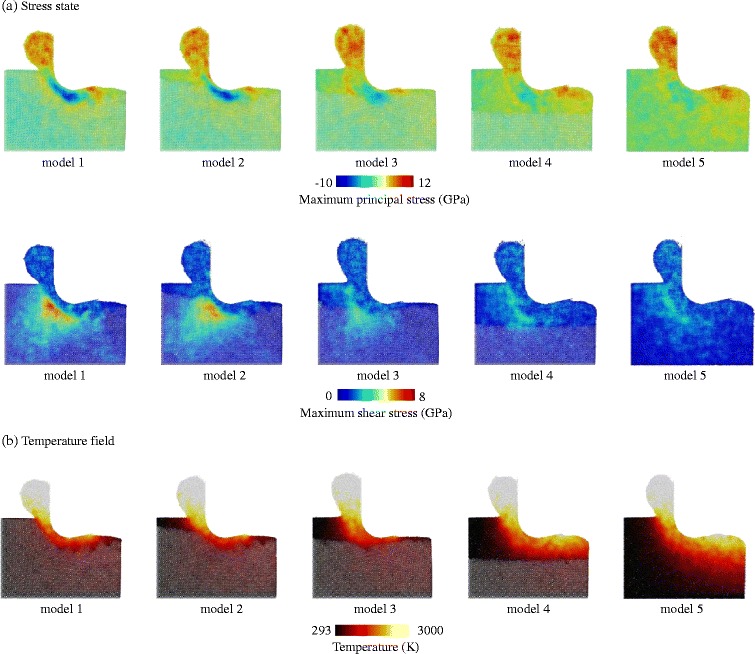
Stress and temperature fields of model-1 ~ model-5

The atomic strain is used to characterize the material deformation [21]. As shown in Fig. 6, there are three distinct regions (denoted by I, II, and III) with strong strain occurring in the workpiece. Two of these regions are located at the rake and clearance faces of the tool, and the other region is ahead of the tool edge. As the amorphous layer becomes thicker, the atomic shear is weakened, especially in region III. To obtain further knowledge of material deformation, the stress fields are computed according to the method employed in the literature [22]. In this work, the maximal principal and maximal shear stresses are used to describe the stress state as shown in Fig. 7a. For c-Si, the material in the vicinity of the tool edge behaves like a fluid due to the high compression indicated by the negative value of the maximal principal stress, while the material out of the compression region maintains its hardness and stiffness. As the fluid phase flows to form the chip, the violent shear strain due to the large velocity gradient occurs on the boundary (i.e., the region III) between the fluid phase and the crystalline bulk. At the same time, the shear stress concentrates in the substrate because of the friction on the phase boundary and the high stiffness of the diamond lattice of c-Si. The lattice structure of the fluid phase is similar to that of a-Si, where the deformability is much stronger than the crystalline phase. Consequently, the stress intensity drops in the amorphous layer. In addition, due to the plasticity enhancement, many more atoms around region III undergo plastic deformation. Then, the velocity gradient and atomic strain are reduced. Apart from the lattice structure, the thermal softening is another mechanism of the plasticity enhancement in a-Si. Amorphous silicon has a lower thermal conductivity than its crystalline phase, which leads to a higher temperature rise, as shown in Fig. 7b.

**Fig. 8:**
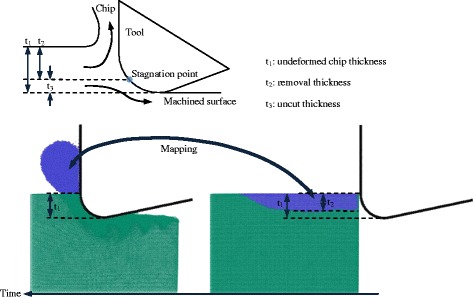
Uncut thickness evaluation

**Fig. 9:**
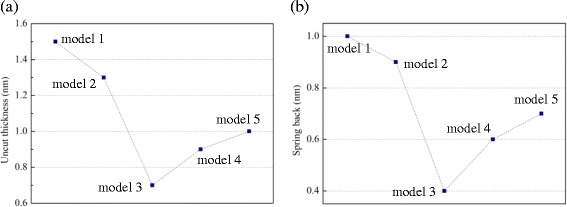
Uncut thickness (**a**) and spring back (**b**) of model-1 ~ model-5

Another region with a slight shear strain denoted by “IV”, as shown in Fig. 6, indicates the existence of the stagnation point where the branching of the plastic flow occurs [23]. Material above this point would flow to form the chip, while material under this point would be depressed under the tool. As a result, a part of the material with a so-called uncut thickness would not be removed. To extract the uncut material, atoms could be colored according to their coordinate in the *y* direction [24]. In this paper, another skill is adopted, as illustrated in Fig. 8. By mapping the atoms in the chip back to the initial state, the uncut thickness can be obtained through the undeformed chip thickness subtracted from the removal thickness. As shown in Fig. 9a, the uncut thicknesses of the a-c and a-Si models are less than the uncut thickness of c-Si, which also implies a larger material removal rate. In particular, this removal rate reaches a minimum when the thickness of the a-Si layer is equal to the undeformed chip thickness. Interestingly, as shown in Fig. 9b, the same tendency is observed for the spring back, indicating the elastic recovery. Small spring back means a low shape error, which has a great value for the ultra-precision machining. Therefore, an amorphous layer with this special thickness is preferred.

**Fig. 10:**
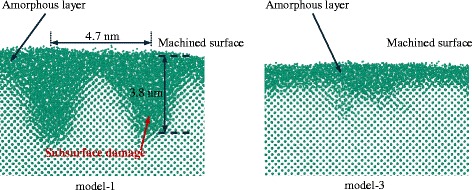
Subsurface damage in model-1 and model-3

In addition, the subsurface damages beneath the machined surface are attenuated by the amorphous layer. As shown in Fig. 10, in the c-Si model, the periodic lattice damages are left under the top amorphous layer as the tool passes by. The damage depth of approximately 3.8 nm is of the same order as the undeformed chip thickness of 5 nm, while there is only a thin a-Si layer on the top of the machined surface in model-3. Thus, once the amorphous layer has the thickness of the undeformed chip, the penetration of the subsurface damage can be hindered, and a desirable machinability on the nanoscale would be achieved.

### c-a-c Models

**Fig. 11:**
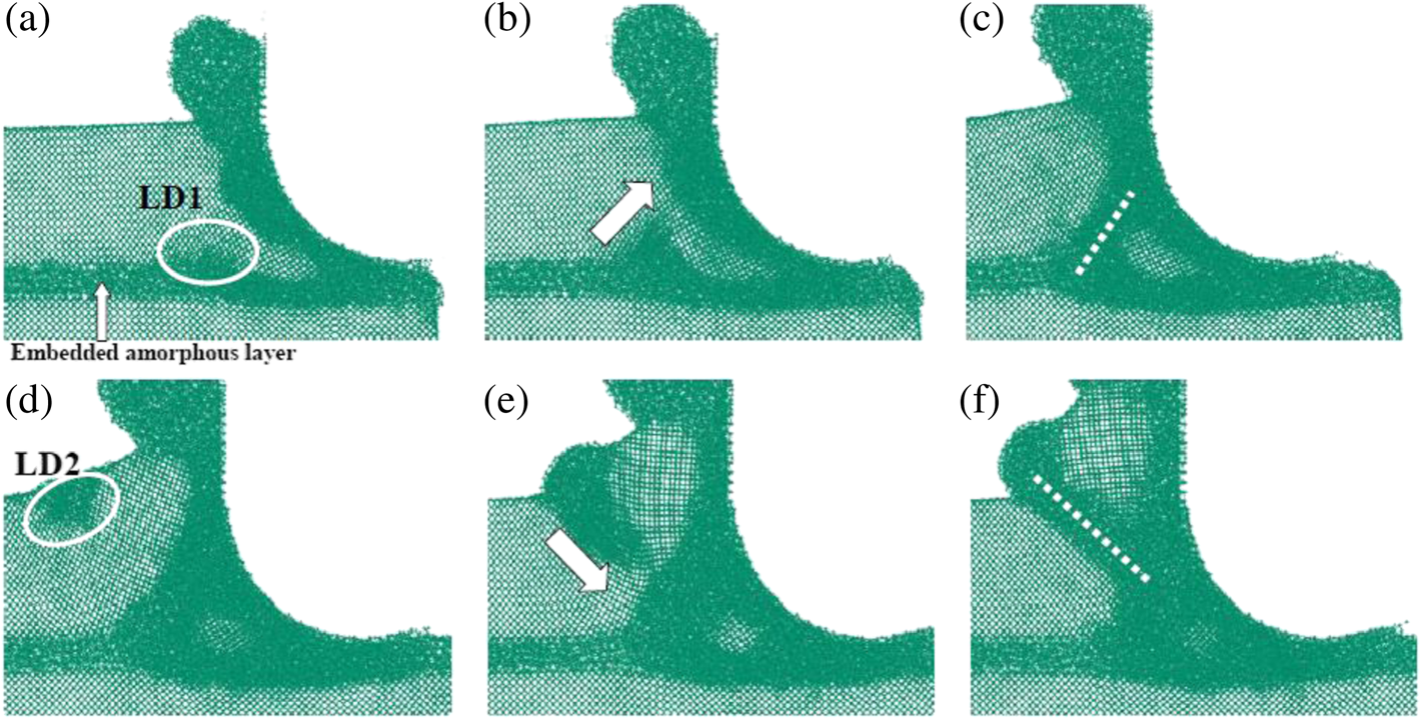
Local deformation band during nanometric cutting on model-6﻿ (**﻿﻿a**) Local deformation formed in the amorphous layer (LD1). (**b**) Extension of the LD1 to the tool edge. (**c**) Formation of a deformation band. (**d**) Local deformation formed in the free surface (LD2). (**e**) Downward extension of the LD2. (**f**) Formation of another deformation band (shear band)

As a thin amorphous layer is located just beneath the tool edge, a local deformation (LD1) is initiated from the amorphous layer, as shown in Fig. 11a. This deformation region then extends upward and merges with the amorphous region around the tool (Fig. 11b, c). With the formation of the new deformation band, the crystalline part above the amorphous layer undergoes obvious elastic bending. As a result, another local deformation region (LD2) occurs from the free surface to relax the high elastic strain, and this local deformation region propagates downward into the material along the easy slip direction of c-Si (Fig. 11d, e). Finally, the local deformation band is formed, and a large piece of material is removed, as shown in Fig. 11f.

**Fig. 12:**
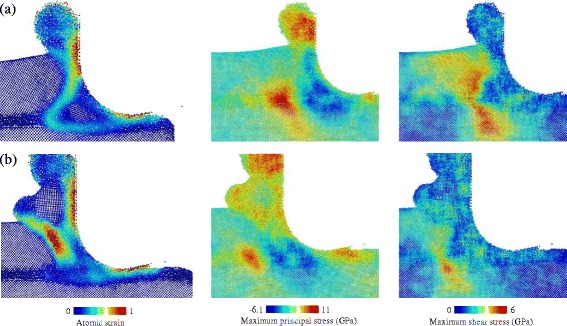
Atomic strain and stresses of model-6. **a** and **b** correspond to the (c) and (f) in Fig. 11

Clearly, the occurrence of the local deformation regions (LD1 and LD2) is critical for the nontrivial phenomena described above. As shown in Fig. 12a, the evolution of the LD1 results in a wedge-shaped deformation front with high tensile stress ahead of its round tip and the shear stress, indicating the elastic deformation in the crystalline phase, very similar to the Griffith model of crack propagation in the stable mode [25]. The wedge-shaped region splits the workpiece along the thin amorphous layer and bends the c-Si upon it. When the c-Si reaches its elastic limit, plastic deformation (LD2) occurs on the free surface. More importantly, the behavior of the LD2 is the same as the mechanism of the serrated chip formation presented by Shaw [26]. In Fig. 12b, a localized shear band is formed, and the chip with the serrated shape can be observed. The amorphous layer embedded into the subsurface induces periodic formation and evolution of the local deformation regions, which also leads to a mixture of the chip formation mechanism with extrusion and shear.

**Fig. 13:**
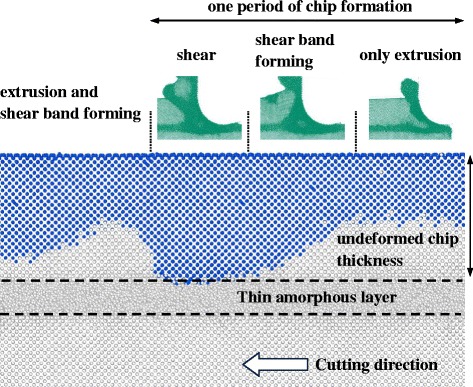
Chip atoms mapped back to the initial configuration of model-6

After the chip atoms are mapped back to the initial state (the blue points in Fig. 13), it shows that the uncut thickness (or the material removal rate) varies during the cutting. Before the formation of the shear band, the extrusion mechanism dominates the chip formation. At this stage, only half of the material is removed. The material removal increases with the extension of the LD2 (i.e., the local shear band). However, in the shear stage, almost all the atoms in the range of the undeformed chip thickness can be removed. As a result, the material removal rate changes periodically as well. Although part of the material is removed by shear, chip formation by extrusion always occurs during the whole nanometric machining operation. In addition, there is no severe subsurface damage, and the spring back is only 0.2 nm.

**Fig. 14:**
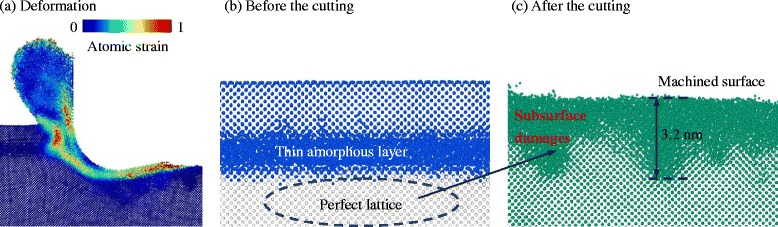
Deformation (**a**), chip atom distribution (**b**), and subsurface damage (**c**) of model-7. The *blue points* in (**b**) would move to form the chip during the cutting

In the model-7, where the depth of the thin amorphous layer is smaller than the undeformed chip thickness, there is no nontrivial phenomenon related to the material deformation and stress state. The chip is formed by extrusion, and the deformation region is merely distorted slightly by the amorphous layer, as shown in Fig. 14a. However, only the c-Si above the a-Si layer is removed (in Fig. 14b), which indicates an 8.6% decrease in the material removal rate compared with monocrystalline silicon model. Besides, the subsurface damage (~3.2 nm deep) is at the same level as c-Si (in Fig. 14c). Therefore, the configuration of model-7 is not desirable.

**Fig. 15:**
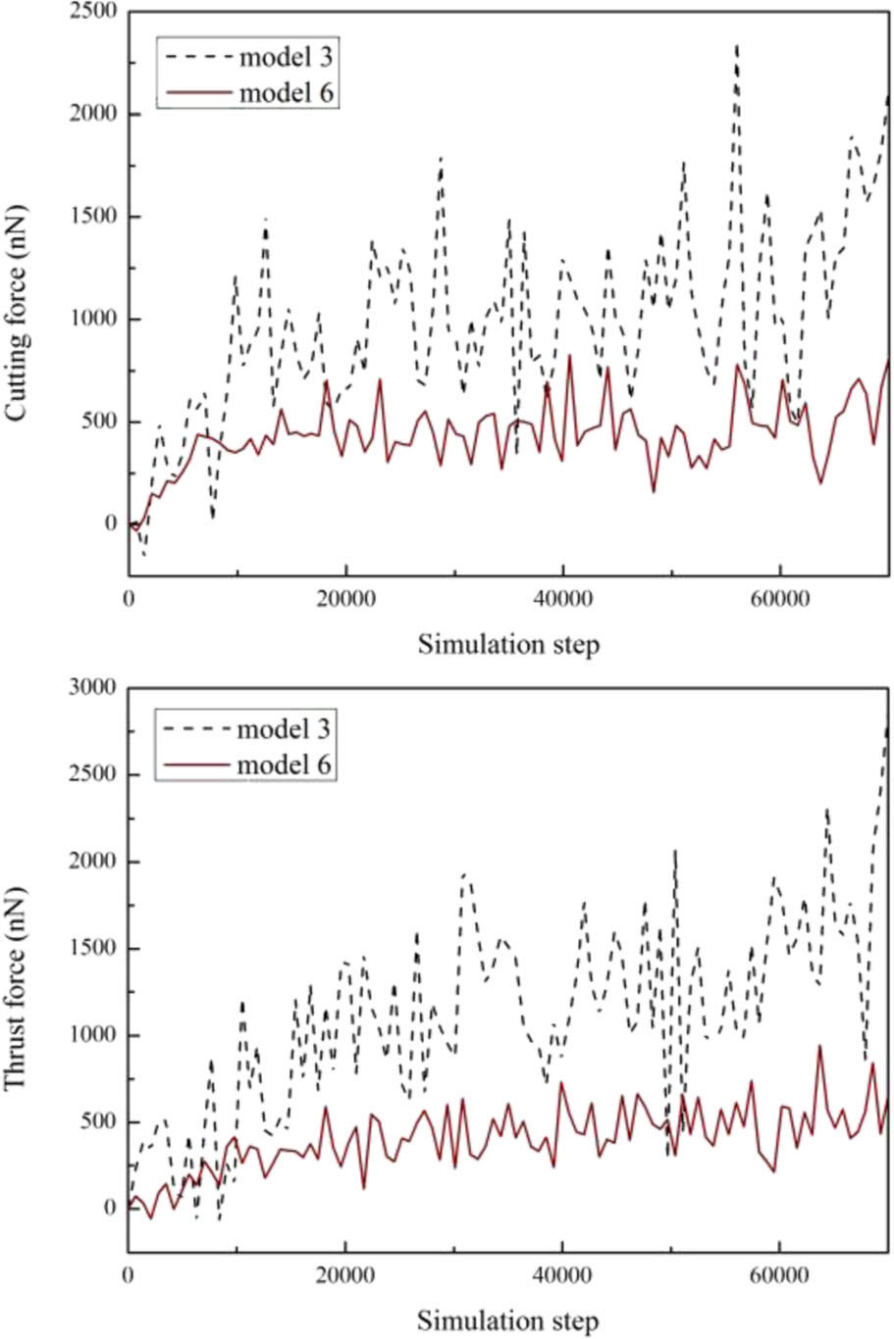
Machining forces of model-3 and model-6

In this study, both the model-3 and the model-6 show a positive effect such as low subsurface damage and small spring back of the amorphous layer on nanometric machining. Nevertheless, the model-6 presents a lower cutting force (in the *x* direction) and thrust force (in the *y* direction), as shown in Fig. 15, which could prolong the tool life significantly. If the amorphous layer is formed by ion implantation, the c-a-c structure in the model-6 would consume very few ions compared to the amorphous layer in the model-3.

## Conclusions

In this paper, the molecular dynamics method is used to study the effect of the amorphous layer on nanometric cutting. To achieve stable models with an amorphous phase, the ensemble conversion should be handled carefully. Adequate length scales of the model in each dimension are essential to depress the artificial effect of the periodic boundary condition. During the ensemble conversion, the continuations of coordinates and velocities should be kept. Some other manners such as the control of the thermal bath are also recommended. The material deformation, stress state, and chip formation are analyzed, and the main conclusions are summarized as follows.

1. For the amorphous-crystalline (c-a) structure, the plastic deformability is enhanced with the increase in the a-Si portion, and stresses are reduced significantly, which has an advantage for the ductile machining of brittle materials. The chip is formed by extrusion independent of the amorphous layer thickness. However, as the a-Si layer extends to the lowest point of the tool, the subsurface damage can be eliminated.
2. With a thin amorphous layer just beneath the tool edge (c-a-c), the chip formation is realized by extrusion together with shear. Periodicities are observed in both the material removal rate and the formation of the local deformation. The local shear band is formed due to the crack tip-like deformation front. The chip formation in this extrusion-shear mixed manner is sensitive to the location of the thin amorphous layer because there is no shear except for in the model-6.
3. In this work, both the a-c (model-3) and c-a-c (model-6) models with specific configurations have low subsurface damage, small spring back, and high material removal rate during nanometric cutting. In addition, a distinct decrease in the cutting forces in the c-a-c structure shows a great value for prolonging the life of the diamond tool. Therefore, surface treatments such as ion implantation can be conducted preceding the mechanical processes to modify the material structure at the nanoscale for a better machinability.
